# Seroprevalence of anti-SARS-CoV-2 among blood donors in Rio de Janeiro, Brazil

**DOI:** 10.11606/s1518-8787.2020054002643

**Published:** 2020-07-01

**Authors:** Luiz Amorim, Célia Landmann Szwarcwald, Sheila de Oliveira Garcia Mateos, Antonio Carlos Monteiro Ponce de Leon, Roberto de Andrade Medronho, Valdiléa Gonçalves Veloso, Josiane Iole França Lopes, Luis Cristovão de Moraes Sobrino Porto, Alexandre Chieppe, Guilherme Loureiro Werneck, Grupo Hemorio de Pesquisa em Covid-19

**Affiliations:** I Hemorio Rio de JaneiroRJ Brasil Hemorio. Rio de Janeiro , RJ , Brasil; II Fundação Instituto Oswaldo Cruz Instituto de Comunicação e Informação Científica e Tecnológica em Saúde Rio de JaneiroRJ Brasil Fundação Instituto Oswaldo Cruz . Instituto de Comunicação e Informação Científica e Tecnológica em Saúde . Rio de Janeiro , RJ , Brasil; III Universidade de São Paulo Faculdade de Medicina da Universidade de São Paulo São PauloSP Brasil Universidade de São Paulo . Faculdade de Medicina da Universidade de São Paulo . São Paulo , SP , Brasil; IV Universidade do Estado do Rio de Janeiro Instituto de Medicina Social Rio de JaneiroRJ Brasil Universidade do Estado do Rio de Janeiro . Instituto de Medicina Social . Rio de Janeiro , RJ , Brasil; V Universidade do Federal do Rio de Janeiro Faculdade de Medicina Rio de JaneiroRJ Brasil Universidade do Federal do Rio de Janeiro . Faculdade de Medicina . Rio de Janeiro , RJ , Brasil; VI Fundação Instituto Oswaldo Cruz Instituto Nacional de Infectologia Evandro Chagas Rio de JaneiroRJ Brasil Fundação Instituto Oswaldo Cruz . Instituto Nacional de Infectologia Evandro Chagas . Rio de Janeiro , RJ , Brasil; VII Universidade do Estado do Rio de Janeiro Laboratório de Histocompatibilidade e Criopreservação, Policlínica Piquet Carneiro Rio de JaneiroRJ Brasil Universidade do Estado do Rio de Janeiro . Laboratório de Histocompatibilidade e Criopreservação, Policlínica Piquet Carneiro . Rio de Janeiro , RJ , Brasil; VIII Secretaria de Estado do Rio de Janeiro Rio de JaneiroRJ Brasil Secretaria de Estado do Rio de Janeiro . Rio de Janeiro , RJ , Brasil; IX Universidade do Federal do Rio de Janeiro Instituto de Estudos em Saúde Coletiva Rio de Janeiro Brasil Universidade do Federal do Rio de Janeiro . Instituto de Estudos em Saúde Coletiva . Rio de Janeiro , Brasil

**Keywords:** Coronavirus Infections, immunology, Blood Donors, Serologic Tests, Seroepidemiologic Studies

## Abstract

**OBJECTIVE:**

To estimate the seroprevalence of antibodies to SARS-CoV-2 among blood donors in the state of Rio de Janeiro, Brazil.

**METHODS:**

Data were collected on 2,857 blood donors from April 14 to 27, 2020. This study reports crude prevalence of antibodies to SARS-CoV-2, population weighted prevalence for the state, and prevalence adjusted for test sensitivity and specificity. Logistic regression models were used to establish the correlates of SARS-CoV-2 prevalence. For the analysis, we considered collection period and site, sociodemographic characteristics, and place of residence.

**RESULTS:**

The proportion of positive tests for SARS-Cov-2, without any adjustment, was 4.0% (95%CI 3.3–4.7%), and the weighted prevalence was 3.8% (95%CI 3.1–4.5%). We found lower estimates after adjusting for test sensitivity and specificity: 3.6% (95%CI 2.7–4.4%) for the non-weighted prevalence, and 3.3% (95%CI 2.6–4.1%) for the weighted prevalence. Collection period was the variable most significantly associated with crude prevalence: the later the period, the higher the prevalence. Regarding sociodemographic characteristics, the younger the blood donor, the higher the prevalence, and the lower the education level, the higher the odds of testing positive for SARS-Cov-2 antibody. We found similar results for weighted prevalence.

**CONCLUSIONS:**

Our findings comply with some basic premises: the increasing trend over time, as the epidemic curve in the state is still on the rise; and the higher prevalence among both the youngest, for moving around more than older age groups, and the less educated, for encountering more difficulties in following social distancing recommendations. Despite the study limitations, we may infer that Rio de Janeiro is far from reaching the required levels of herd immunity against SARS-CoV-2.

## INTRODUCTION

In December 2019, several cases of severe pneumonia of unknown etiology emerged in Wuhan, China. Within a short period after the first case was reported, the outbreak gradually spread across the country and the globe. The causative agent was a betacoronavirus – SARS-CoV-2 –, which elicits a severe acute respiratory syndrome (SARS) called covid-19 ^1^ .

The infectious disease spread rapidly, reaching virtually every country in the world. By the end of the first week of May 2020, there were over 3.8 million confirmed worldwide cases and around 260,000 deaths ^2^ By May 6 ^th^ , Brazil had reported over 125,000 confirmed cases and 8,536 deaths, and a case fatality rate around 7% ^3^ . In Rio de Janeiro, the first case was reported on March 1 ^st^ , 2020. By May 6 ^th^ , the state had 13,295 confirmed cases, 1,205 deaths and a 9.1% fatality rate ^3^ .

The infection often causes mild symptoms, including cough, muscle pain, and anosmia, and it can progress into high fever, pneumonia, respiratory distress ^4^ and, in some cases, death ^5 - 7^ . Yet, in most cases, individuals have few or no symptoms, being a substantial source of transmission and posing a challenge to prevent disease dissemination ^8^ .

Reverse transcription polymerase chain reaction (qRT-PCR) is considered the gold standard technique for detecting and confirming covid-19 ^9^ . However, some studies show a high rate of false-negative tests due to some factors that can influence the results, such as: type of biological sample, inadequate collection, fluctuation of viral load, and the period between blood collection and symptom onset ^10^ . Thus, by performing serological tests we may investigate the presence of acute-phase (IgM) or memory (IgG) antibodies. To facilitate the control of viral transmission and ensure timely public health intervention, it is essential to adopt a simple, sensitive, and specific test, which guarantees immediate and accurate results for promptly identifying SARS-CoV-2-infected patients ^11^ .

It is relevant to know the prevalence of SARS-CoV-2 among asymptomatic people for two major reasons. First, healthy individuals in epidemic areas may be infected and asymptomatic and still represent a significant source of transmission. At the beginning of the epidemic in China, about 86% of infections were not detected, but they were the source of infection for about 79% of the cases ^8^ . Second, herd immunity indicates an infection spread within a community. By monitoring its level, we may owe a reference for guiding future decisions on the right time to start relaxing social distancing measures, minimizing possible subsequent epidemic outbreaks ^12^ .

The seroprevalence of SARS-CoV-2 in asymptomatic groups has been addressed by few studies, among each a major one is the report from the Diamond Prince cruise ship. After an outbreak during the cruise, Japanese health authorities tested 3,063 passengers by RT-PCR and the estimated asymptomatic proportion among all infected cases was 17.9% ^13^ . A study conducted in the county of Santa Clara, California, USA, found a 2.8% seroprevalence of SARS-CoV-2, after adjusting for test sensitivity and specificity and population demographics ^14^ .

Evaluating the trends in the prevalence of viral infections in blood donors is essential not only for estimating the effectiveness of strategies for blood safety, but also for enhancing them, reducing the potential risk of infection by blood transfusion ^15^ . Determining the prevalence of SARS-CoV-2 in blood donors enable the monitoring of the virus circulation among healthy people, helping to implement strategies to reduce transmission, especially in the absence of seroprevalence surveys. Yet, there are but few studies on the prevalence in blood donors. Two of them, sill unpublished, reported 1.7% seroprevalence in blood donors in Denmark, and 2.7% in the Netherlands ^16 , 17^ .

During the final two weeks of April 2020, we conducted a seroprevalence survey among volunteer blood donors of Hemorio, the main blood center in Rio de Janeiro, Brazil. This manuscript reports the prevalence of antibodies to SARS-CoV-2 within a sample of 2,857 volunteer blood donors, adjusting for gender and age group to supply such information to health authorities for estimates, extrapolations, and health interventions. To date, this is the first study in Latin America addressing the seroprevalence of SARS-CoV-2 in asymptomatic blood donors.

## METHODS

### Study Design

Cross-sectional study consisting of serological testing in volunteer blood donors. For the analysis, we considered sociodemographic data – age, gender, donation site (fixed or mobile donation sites) – education level, and place of residence (within the capital or other municipalities of Rio de Janeiro).

The donor management software (SACS) of the Blood Center provided individuals’ demographic data using a code, without their identification. The study group is formed by the total number of people who donated blood in the Hemorio Blood Center from April 14 ^th^ to April 27 ^th^ .

### Study Subjects

In Brazil, before blood donation, candidates had to complete a written questionnaire and undergo a brief health screening. For candidates to be accepted as blood donors in Hemorio, they had to comply with all the donation eligibility criteria set by the Brazilian Ministry of Health and the American Association of Blood Banks ^18^ . Recently, some criteria regarding covid-19 have been included: prospective donors could not have had flulike symptoms within the 30 days before donation; had close contact with suspected or confirmed covid-19 cases in the 30 days before donation; or traveled abroad in the past 30 days. Candidates presenting fever (forehead temperature > 37.8 ^o^ C) on the donation date are also rejected. Thus, individuals in the study group had no symptoms of covid-19 and no known historical epidemiology of the disease.

Once accepted to donate blood, they were automatically included in the study, provided they agree to sign the informed consent form for blood donation and testing for other pathogens – not included within the infectious diseases markers required to be tested in all blood donations in Brazil. Both blood donation and sample collection were performed at a fixed donation site, Hemorio’s facilities, or at mobile sites, in churches and private condominiums, in Rio de Janeiro.

This study was approved by the Research Ethics Committee of the Hemorio – (Approval No: 4.008.095).

All individuals classified as eligible for donation during the study period participated in the survey. We excluded those who refused to sign the informed consent form for blood donation and testing.

### Sample Collection

The serum used for testing infectious disease markers were also used for SARS-Cov-2 antibody test. At the beginning of blood donation, we collected and barcoded those samples for each donor.

### Antibody Testing

To detect IgG and IgM anti-SARS-CoV-2 antibodies, we performed the rapid test MedTest Coronavirus 2019-nCoV IgG/IgM, from MedLevensohn manufacturer (Yuhang District, China): an immunochromatographic assay which combines SARS-COV-2 antigen-coated particles to qualitatively detect IgG and IgM antibodies. The MedTest Coronavirus (covid-19) IgG / IgM, licensed by the Brazilian Health Surveillance Agency (ANVISA) in March 2020 (https://consultas.anvisa.gov.br/#/saude/q/?numeroRegistro=80560310056), can detect SARS-CoV-2 antibodies in whole blood, capillary blood, serum, and plasma. We performed the tests with serum, following the manufacturer’s instructions.

### Real-Time Polymerase Chain Reaction (RT-PCR tests)

We tested serum or plasma from antibody-positive samples (IgM, IgG, or IgG + IgM) to detect SARS-CoV-2 by qRT-PCR – (Molecular IDT IntegratedDNA TechnologiesSARS-CoV-2 – N1/N2/P, Promega, Madison, USA).

For RNA extraction, we used MDX Instrument from Qiagen (Hilden, Germany) and Applied Biosystem MDX thermocycler instrument, from Thermo-Fisher (Waltham, USA), following the manufacturer’s instructions.

### Statistical Analysis

We tabulated the data in an Excel® spreadsheet with donors demographic characteristics reported by code, so that their individual identity would be anonymous.

The prevalence of covid-19 in the population was measured by three steps. First, we reported the crude rates of positive tests without adjustments. Second, we estimated the weighted prevalence using Rio de Janeiro population in 2020. This adjustment was necessary to balance our sample based on population distribution according to gender and age. Third, we adjusted the prevalence for test sensitivity at 85% and specificity at 99%, following the manufacturer’s estimates. The true or adjusted prevalence and its 95% confidence interval were set using a previously published estimate ^19^ .

For statistical analysis, we considered two outcomes: the unadjusted and weighted prevalence of the test for antibodies to SARS-Cov-2. The following variables were also considered: gender, age group (18-29; 30-49; 50+), donation site (Hemorio, churches, condominiums), education level (higher education; secondary education) and place of residence (within the capital or other municipalities in of Rio de Janeiro). To investigate a possible increasing trend, the collection dates were framed into three periods: April 14 ^th^ to 18 ^th^ ; April 19 ^th^ to 23 ^rd^ ; and April 24 ^th^ to 27 ^th^ .

To establish the correlates of SARS-CoV-2 infection, we used logistic regression models and odds ratio (OR). Statistical tests at 5% significance level were adopted for relating the prevalence of antibodies to SARS-Cov-2 (IgG, IgM or IgG+IgM) to donors’ characteristics (gender, age group, educational level, place of residence, and donation site and period).

Statistical analysis was performed using version 12 STATA (STATA Corp., College Station, Texas, USA).

## RESULTS

### Antibody Testing

The study sample was composed by 2,857 volunteer blood donors, all of which were tested for IgG and IgM anti-SARS-CoV-2. The overall prevalence of antibody was 4%; Tables 1, 2, and 3 show these results in detail.

**Figure 1:**
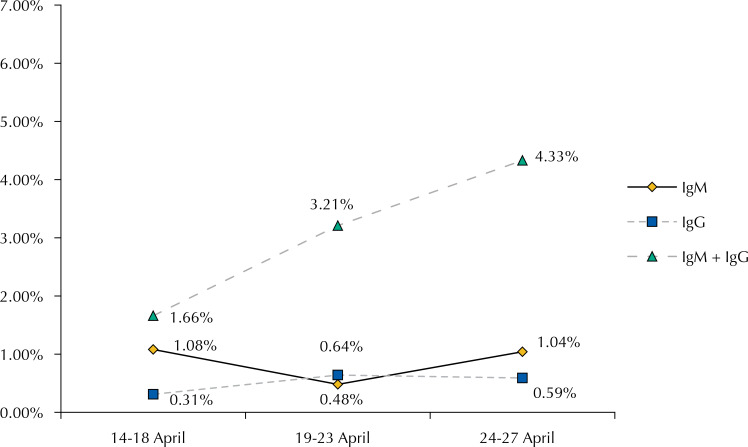
Prevalence by period of time according to the type of antibody detected.

Regarding the type of antibody detected, IgM-only comprised 23.7% of positive results, IgG-only 11.4%, and IgM+IgG 64.9%. Figure 1 shows the prevalence rates according to period (April 14-18 ^th^ , April 19-23 ^rd^ , and April 24-27 ^th^ ).

Table 1 shows four prevalence estimates. The prevalence of SARS-Cov-2 positive tests without adjustments (crude prevalence) was 4.0% (95%CI 3.3–4.7%). The weighted prevalence according to the population of Rio de Janeiro was slightly lower (3.8%; 95%CI 3.1–4.5%). Further adjustment for test sensitivity and specificity resulted in even lower estimates: 3.6% (95%CI 2.7–4.4%) for the non-weighted prevalence, and 3.3 (95%CI 2.6–4.1%) for the weighted prevalence.

**Table 1:** Seroprevalence of antibodies to SARS-Cov-2 in blood donors, estimates adjusted for specificity and sensitivity. Rio de Janeiro, Brazil, April 14-27, 2020.

Estimates	Sample size	Prevalence (%)	95%CI
Unadjusted	2,857	4.0	3.3–4.7
Weighted* for Rio de Janeiro population	2,857	3.8	3.1–4.5
Adjusted for sensitivity and specificity	2,857	3.6	2.7–4.4
Weighted* estimate adjusted for sensitivity and specificity	2,857	3.3	2.6–4.1

* Weighted according to the population of Rio de Janeiro aged 18-69 years, by age and gender.

In the logistic regression analyses ( Table 2 ), some of the covariates were significantly associated with the crude prevalence of antibodies to SARS-Cov-2. Collection period was the variable most significantly associated with the crude prevalence: the later the period, the higher the prevalence. In the third period (April 24-27 ^th^ ) the chances of positive test for SARS-Cov-2 antibodies was twice as high as in the first period (April 14-18 ^th^ ) (OR = 2.05; 95%CI 1.33-3.16). Regarding sociodemographic characteristics, the younger the blood donors, the higher the prevalence; and the lower the education level, the higher the chances of testing positive for antibodies response to SARS-Cov-2. We found no statistically significant difference for gender and place of residence (capital or elsewhere). Collection site was also significantly associated with the crude prevalence: blood donors from condominiums showed a significantly lower prevalence than blood donors from Hemorio.

**Table 2:** Unadjusted seroprevalence of antibodies to SARS-Cov-2 in blood donors according to donor’s characteristics. Rio de Janeiro, Brazil, April 14-27, 2020

Variables		Sample size	Prevalence (%)	OR	95%CI	p
Gender	M	1,450	4.2	1.12	0.77–1.63	0.548
	F	1,407	3.8	1.00	-	-
Age group	18–29	870	5.2	1.80	1.01–3.22	0.047*
	30–49	1,443	3.7	1.26	0.71–2.22	0.428
	50–69	544	2.9	1.00	-	-
Education level	No higher education	1,753	4.7	1.72	1.13–2.62	0.011*
	Higher education	1,104	2.8	1.00	-	-
Period	April, 14–18	1,565	3.0	1.00	-	-
	April 19–23	623	4.3	1.46	0.90–2.37	0.122
	April, 24–27	669	6.0	2.05	1.33–3.16	0.001*
Place of residence	Capital	2,090	3.8	0.86	0.57–1.29	0.464
	Other municipalities	767	4.4	1.00	-	-
Donation site	Churches	820	3.8	0.81	0.53–1.24	0.325
	Condominiums	466	2.1	0.45	0.23–0.88	0.019*
	HEMORIO	1,571	4.6	1.00	-	-

* 5% significance level.

We found similar results for the weighted prevalence of antibodies to SARS-Cov-2 ( Table 3 ). The variables found to be significantly associated to the crude prevalence were also significantly associated with the weighted prevalence. However, by weighting the sample, we found a more accentuated statistical significance for the 18-29 age group (OR = 1.86; 95%CI 1.12–3.08%), for lower education level individuals (OR = 2.11; 95%CI 1.35–3.28), and for condominium donors (OR = 0.45; 95%CI 0.23–0.86%). Collection period was also significantly associated to the weighted prevalence (p < 0.005), but OR was a little higher for the crude prevalence.

**Table 3:** Weighted a seroprevalence of antibodies to SARS-Cov-2 in blood donors according to donor’s characteristics. Rio de Janeiro, Brazil, April 14-27, 2020

Variables		Sample size	Prevalence (%)	OR	95%CI	p
Gender	M	1,387	4.1	1.20	0.82–1.76	0.352
	F	1,470	3.5	1.00	-	-
Age group	18–29	718	5.3	1.86	1.12–3.08	0.015 ^b^
	30–49	1,199	3.6	1.26	0.77–2.04	0.357
	50–69	940	2.9	1.00	-	-
Education level	No higher education	1,722	4.8	2.11	1.35–3.28	0.001 ^b^
	Higher education	1,135	2.3	1.00	-	-
Period	April, 14–18	1,549	2.8	1.00	-	-
	April 19–23	624	4.5	1.60	0.98–2.58	0.058
	April, 24–27	684	5.3	1.91	1.22–2.99	0.005 ^b^
Place of residence	Capital	2,110	3.7	0.92	0.60–1.41	0.688
	Other municipalities	747	4.0	1.00	-	-
Donation site	Churches	800	3.6	0.80	0.51–1.24	0.313
	Condominiums	515	2.1	0.45	0.23–0.86	0.016 ^b^
	Hemorio	1,542	4.5	1.00	-	-

^a^ Weighted according to Rio de Janeiro population aged 18-69 years, by age and gender.

^b^ 5% significance level.

### qRT-PCR Tests

We tested all the antibody-positive samples – IgG and/or IgM – by PCR, and found no PCR-positive test among them.

## DISCUSSION

In a survey on antibodies responses for SARS-CoV-2 among Brazilian blood donors, we found a seroprevalence of 3.3% (95%CI 2.6–4.1), adjusted for test sensitivity and specificity and weighted according to the population of Rio de Janeiro aged from 18 to 69 years, by age group and gender. This estimate is higher than that observed in two seroprevalence surveys among blood donors, conducted in Denmark and the Netherlands (1.7% and 2.7%, respectively) ^16 , 17^ . The prevalence varied substantially among subgroups: the youngest and less educated presented significantly higher values. We also found an increasing linear trend in the prevalence along the study period: 2.8% during the first week, 4.5% during the second, and 5.3% during the third (p < 0.01); resulting mainly from the increase in IgG + IgM antibodies.

Two months after the first covid-19 case in Rio de Janeiro, over 13,000 confirmed cases and 1,000 deaths had been reported ^3^ . In the early weeks of March, the state adopted several measures for restricting social interaction and improving diagnostic capacity ^20^ yet, the epidemic curve is still on the rise and hospital services for covid-19 care face an imminent collapse ^21^ .

The questions of whether and when such measures should be implemented or strengthened have played a leading role on debates held among public health researchers and professionals, health authorities, and communities. A feasible guide for such decisions is the level of herd immunity within a population: levels around 60% have been considered the threshold for the disease, based on the available estimates of the basic reproduction number of the infectious agent ^22^ . For the lack of vaccine against the covid-19, such level of herd immunity would only be achieved by natural infection. However, in settings such as Rio de Janeiro, in which a forthcoming breakdown of the health care system is expected, fostering natural herd immunity is an unreasonable option – it would require relaxing the social distancing measures, what would increase the number of deaths by covid-19. Conversely, the effectiveness and length of such measures will decrease the capacity of achieving natural herd immunity, impair the implementation of exit strategies, and increase the risk of future epidemic outbreaks ^23^ .

Our results indicate that achieving an effective level of herd immunity would be challenging in the short-term. Thus, relaxing social distancing measures might be unwise in the immediate horizon and must be carefully pondered in the future while considering infrastructure availability in hospitals – particularly ICU beds and ventilators, which provide the appropriate care for severe covid-19 patients. It is unclear whether the neutralizing antibody response provides the required effect for preventing new infections ^24^ . In case just a fraction of the individuals presenting antibodies shows neutralizing antibodies, then the target herd immunity level would have to be increased. In these circumstances, the desired level of herd immunity will most likely not be achieved before an effective vaccine becomes available.

We believe this study comprises the first large seroprevalence survey for SARS-CoV-2 infection in asymptomatic people conducted in Rio de Janeiro, Brazil. The study group is not a random sample, but it accounts for a demographically and socially heterogeneous healthy population, allowing a preliminary outlook of the prevalence of the antibody in asymptomatic individuals. Our estimates were adjusted for test sensitivity and specificity and weighted by population age and gender, providing a better view of the prevalence of the antibody at a population level.

Our results corroborate some basic premises. We found an increasing (and already expected) seroprevalence over time, given that the epidemic curve has been on the rise for the past two months in Rio de Janeiro, without any sign of decreasing ^21^ . The higher prevalence of the antibody among the youngest was also predictable, as they comprise the core workforce and are more likely to move around, being exposed to the infection even under social distancing restrictions. Likewise, we expected a higher prevalence among the less educated, as they often pertain to lower socio-economic stratum and encounter greater difficulties in following social distancing recommendation for having to look for some source of income. Many of them also live in crowded households, without piped water, hindering the adoption of basic hygiene measures. A study conducted in the state of Ceará found that individuals with primary education considered themselves at lower risk for getting covid-19 and were less engaged in voluntary quarantine than those with higher education levels ^25^ . At last, we also anticipated that blood donors from condominiums would present lower prevalence, as the donation site is right at their living place, which suggests that they follow social distancing recommendations. Conversely, those donating blood at the Hemorio blood center are more likely to do so while coming to the city center for working or other reasons.

This study results should be deemed with caution. The study groups vary in demographic and social terms, but still comprise a convenience sample. Thus, extrapolating the results for the overall population of Rio de Janeiro or even only for those aged between 18 to 69 years might be biased. We did not selected the sample for providing estimates for different regions of the State, but we expect the prevalence of infection to vary across different geographical areas of the city. At last, we adopted values provided by the manufacturer for the adjusted prevalence estimates for sensitivity and specificity, but they might not be valid for the Brazilian population profile. Yet, the specificity value (99%) was confirmed by a pilot study with 120 plasma samples from Hemorio’s blood donor repository, conducted in 2018 – long before the novel Coronavirus pandemic. Among these 120 samples only one tested positive for SARS-CoV-2 antibodies.

Despite the limitations, we may infer that effective levels of natural herd immunity to SARS-CoV-2 are far from being reached in Rio de Janeiro, considering the social distancing implemented measures, and should not be deemed a target for a short-term exit plan. Stipulating the adequate time for relaxing such measures in the short-term should consider the availability of adequate health care infrastructure, until a larger and population-based serological survey could be conducted. Such a survey should aim at identifying the variations in the levels of herd immunity within the state, and eventually recommend a more locally-oriented strategy, considering levels of natural herd immunity, degree of vulnerability of the population, and the availability of adequate resources for testing and treating the severe cases of covid-19.
